# Strong culture, healthy lifestyles: a mixed methods feasibility study for a co-created afterschool cultural programme for Australian Aboriginal children

**DOI:** 10.1186/s40814-023-01422-y

**Published:** 2024-02-15

**Authors:** Rebecca M. Stanley, Anthony McKnight, Yasmine Probst, Gabrielle O’Flynn, Sarah Tillott, Cullunghutti Aboriginal Child and Family Centre

**Affiliations:** 1 Early Start, Faculty of the Arts, Social Sciences and Humanities, University of Wollongong, Wollongong, NSW 2522 Australia; 2 Illawarra Health and Medical Research Institute, Wollongong, NSW 2500 Australia; 3 School of Health and Society, Faculty of the Arts, Social Sciences and Humanities, University of Wollongong, Wollongong, NSW 2522 Australia; 4 School of Education, Faculty of the Arts, Social Sciences and Humanities, University of Wollongong, Wollongong, NSW 2522 Australia; 5 Faculty of Health, Southern Cross University, Gold Coast Campus, Bilinga, QLD 4225 Australia; 6 Cullunghutti Aboriginal Child and Family Centre, Nowra, NSW 2541 Australia

**Keywords:** Indigenous, Australian Aboriginal, Wellbeing, Afterschool, Culture, Child, Feasibility, Relational

## Abstract

**Background:**

Having a strong connection to culture and Country is fundamental to the health and wellbeing of Australian Aboriginal children. The aim of the research was to evaluate the feasibility of study methods and programme implementation of a co-created afterschool cultural programme, and identify areas for improvement.

**Methods:**

Aboriginal Relational Research Methodology and mixed methods were applied to evaluate the feasibility of the implementation of the programme and study methods using a non-randomised single-group study design. Australian Aboriginal children and their siblings aged 5–13 years were recruited within regional New South Wales, Australia. The primary outcomes for feasibility included recruitment rates of children and Aboriginal programme mentors, compliance rates of outcome data collection and of the planned programme activities, programme attendance, retention rates and mean enjoyment scores. Follow-up yarning circles were conducted with the children, their parents/caregivers, programme mentors and teachers to explore aspects of feasibility, and areas for improvement.

**Results:**

A total of 90 caregivers consented to their children (*n* = 111) being part of the research. Sixteen Aboriginal mentors were recruited to deliver the programmes across the communities. Overall, 74.4% of all health outcome measures were completed across baseline (86.5%) and follow-up (55.9%). Only 61.0% of the programme activities were delivered as originally planned. The average programme attendance rate was 70.0% with a 92.0% retention rate. Eighty-nine percent of children reported a high level of enjoyment with the programmes. Follow-up yarning identified the importance of relational methodologies and flexibility within the programme design and implementation to ensure programmes were adapted to the local community, conditions and differing age groups. Considerations for future programmes included the timing of the programme and identifying health outcome assessment tools and methods that acknowledge cultural protocols and experiences.

**Conclusions:**

Engaging the communities in the development, implementation and evaluation of the programmes were key to community support of the programme and conducting the feasibility study. Future programmes and evaluations need to be built on strong partnerships and embrace flexible and culturally embedded methodologies in order to be adaptive and responsive to research approaches, communities and to Country.

**Trial registration:**

ACTRN12619001224112. Retrospectively registered on 05 September 2019.

## Key messages regarding feasibility

1. What uncertainties existed regarding the feasibility?

The uncertainties pertained to realistic targets for recruitment, retention and compliance rates, of a co-created afterschool cultural programme, as well as suitability of study methods and research tools.

(2) What are the key feasibility findings?

The research exposed a disjunction between Western feasibility constructs and understanding feasibility using Aboriginal Relational Research Methodology (ARRM) in the context of an afterschool cultural programme. The results showed that the communities embraced a programme that was adapted to the local context. Potential targets for participation, recruitment, retention and compliance within the programmes were identified. These were favourable due to continual negotiation with the communities and consideration of Country across all stages. A relational methodology, inclusive of mixed methods, was vital to programme acceptance and gaining a deeper understanding of the key findings.

(3) What are the implications of the feasibility findings for the design of the main study?

A “one size fits all” approach is not suited to the implementation of an afterschool cultural programme in other Aboriginal communities. Future programmes need to be respectfully adaptable and responsive to research approaches, the communities and to Country.

## Background

Enhancing health and educational outcomes among Aboriginal children through cultural connectedness can be achieved using a relational community-based intervention. The “Strong Culture, Healthy Lifestyles” project is a participatory community-based project that was initiated between a non-Aboriginal academic researcher (RS) and an Aboriginal woman, the Chief Executive Officer of an Aboriginal Child and Family Centre. The project team have worked together since 2014 to explore how connecting with culture and Country impacts health, lifestyle behaviours and educational outcomes of Aboriginal children and their non-Aboriginal siblings through an afterschool cultural programme. The project evolved into a partnership between *Yuin* Country, Aboriginal community organisations, Aboriginal Elders, community members and academic researchers (both Aboriginal and non-Aboriginal). To respect this partnership, the authors would like to acknowledge the traditional custodians of *Yuin* Country, and their continuing connection to the land, sea and community and pay their respects to the Ancients, Ancestors and the Elders of today and the future.

Country informs the framework of the project. Country is a breathing living entity which people come from and have a custodial role to care for, as Country cares for them [1]. Kwaymullina [2] articulates Country as being “loved, needed, and cared for, and Country loves, needs, and cares for her peoples in turn. Country is family, culture, identity. Country is self” (p. 12). Country provides everything to human beings that is needed to survive, for example, water, air, communication and food. There are, however, many things through Country that connect people, and this is the framework that the project team are working from to identify the connections for the children in the project.

### Country, wellbeing and Indigenous health programmes

Australia has two similar yet distinct cultural groups—Aboriginal and Torres Strait Islander peoples. From this point on, the community have asked the authors to refer to them as Aboriginal peoples. Having a strong connection to culture and Country is fundamental to the health and wellbeing of Aboriginal peoples [3–5]. The concepts of Country, health and wellbeing are perceived as holistic and interconnected, encompassing the social and emotional wellbeing (SEWB), physical, cultural and spiritual health [6–9]. Other authors [10–12] have shown that people’s health is intricately linked to their connection to and their caring of Country. When this connection is disrupted, the health of Aboriginal peoples suffer [10, 11]. For a number of Aboriginal children, the intergenerational cultural, socioeconomic and political impact of colonisation has also meant that they may not be provided with opportunities to engage with and learn about their culture and connect with Country [4, 13], placing their health at risk.

Despite recognition of the importance of culture on health and wellbeing [3–5, 7–9], few programmes have been developed with culture at the core to enhance a person’s physical, SEWB and spiritual health [5, 14, 15]. Even though there is a growing body of literature on strength-based approaches and placing culture and Country centre to SEWB, mainstream approaches to programme development and evaluation for Aboriginal peoples still perpetuate the deficit discourse, focusing on isolated causes of illness, rather than protective factors and strength-based approaches [16]. Viewing health from a holistic and interconnected perspective is vital when including the cultural determinants of health [5, 17]. Furthermore, health interventions and programmes tend to be developed, implemented and evaluated from an “expert” model or top-down approach. These approaches often involve a linear sequence of studies developed and tested under tightly controlled conditions [18], with narrowly defined and measured outcomes. Once efficacy is demonstrated, the programmes are implemented as a “one size fits all” [19], which do not acknowledge the need for flexibility to account for the diversity of each community. The approach is further compounded by the historical nature of Indigenous health research where research focusing on Aboriginal peoples’ health and wellbeing has been conducted *on*, rather than *with* or *by* Aboriginal peoples. These approaches provide limited benefit for the communities and are often damaging and exploitive in nature [20–22].

Recent approaches informed by Aboriginal relational methodologies (for example McKnight [1]) and Indigenous Research Methodology (IRM) [23] challenge the mainstream approaches to health research [22]. IRM is considered a critically reflexive and transformative process of research that incorporates a deep understanding of Indigenous culture [22, 24]. Relational approaches are strongly connected to strength-based research and shift the power and decision-making processes by adopting a co-leadership approach with communities placing a focus on Country. Such approaches are fundamental to both decolonising research about Aboriginal health [25] and to empowering Aboriginal communities. Relational approaches demonstrate the interconnectedness of everything that is Country including people’s health. If one of these relationships is disrupted, everything is, and vice versa [26]. For the purpose of this paper, the authors will refer to their methodology as Aboriginal Relational Research Methodology (ARRM) [26]. ARRM draws on different aspects of Aboriginal relational methodologies, such as oneness [1], co-becoming [27], relationality [21] and relatedness [28]. We have used ARRM to demonstrate the similarities and connectedness to a range of Indigenous authors working in this space to show respect to everyone each time we mention ARRM.

The aim of this study was to evaluate the feasibility of the study methods and implementation of a co-created afterschool cultural programme, specifically recruitment, retention, attendance, health outcome data collection and programme delivery compliance rates and enjoyment, and identify areas for improvement prior to its adaptation into other communities. It is important to point out that ARRM informed how feasibility was understood, defined and classified from an Aboriginal viewpoint. As such, the feasibility was concerned with appraising the relational context and concepts, which is inclusive of the relationships between Country to people, people to Country, people to people, methods to people and how Country informs, for example, the programme and research evaluation. Thereby, a Western understanding of feasibility may be challenged.

## Methods

### Methodology

The ARRM approach for this project was actioned to be more inclusive of Country and community [26]. The methodology required the research team to examine self and positionality in relation to Aboriginal health research methods, to engage Aboriginal communities [23, 29, 30]. The methodological framework consisted of a reciprocal, emotional, negotiated-re-negotiated space, which was constantly evolving and needed to be flexible and adaptable to find points of connection between Western and Aboriginal knowledge systems [31, 32]. A feature of the methodology is the acknowledgment of key concepts and entities that go beyond the human, that connect in oneness the mental, physical and spiritual to guide the direction of the project. The focal concepts and entities within this relationship were Country and Child. For more details about this methodology, please refer to McKnight et al. [26].

### Study design

The overall “Strong Culture, Healthy Lifestyles” project is a non-randomised single-group study (ACTRN12619001224112) of an afterschool cultural programme co-created and delivered by selected Aboriginal people (mentors) to primary school-aged children from the respective communities.

Following Elder approval, meetings were held with key organisations and stakeholders to develop and initiate the project through the ARRM approach. The project has evolved through three phases. The first phase was a needs assessment through Aboriginal community consultations [33]. The second phase was used to develop, implement and evaluate the feasibility of the study methods and the afterschool cultural programme in three New South Wales (NSW) Aboriginal communities. The third phase will apply the lessons learned from the second phase to further explore the feasibility and potential efficacy of implementing an afterschool cultural programme. This paper reports on the second phase.

This current study was designed to identify and evaluate realistic targets such as recruitment, retention and compliance rates, for future studies. For the purpose of this paper, “study” refers to the elements of the research related to the afterschool programmes in each community, while “project” refers to the wider implementation of the afterschool programmes in the region.

### Setting and participants

Three Aboriginal communities from the South Coast region of NSW, Australia, on *Yuin* Country, were identified by community Elders and leaders for this study. The project involved running six programmes across the three communities (two rounds per community). Each community, with the approval from the local Elders, decided the location where the programme would run. The facilities used by the three communities included a local primary school, a local Aboriginal Child and Family Centre, and the third community held the programme at a local Aboriginal Community. All communities took the children on Country to areas of cultural significance within the region such as the river, ocean or bushland. Transportation was provided where necessary. Programmes were provided free of charge to the children who participated.

Children were identified by the local primary schools and were eligible if they were aged 5–13 years old, identified as being Aboriginal and/or Torres Strait Islander, or a sibling of an Aboriginal and/or Torres Strait Islander child. Given this was the first programme of its type to be created and delivered in these communities, no sample size was specified, rather the feasibility trial was used to identify how many children could be recruited to the programme. However, there was a restriction on the upper limit of the number of children per programme based on Australian outside of school hours care guidelines for staff to child ratios (i.e. 1:15) [34]. For our context, this meant mentor to child ratios to achieve our primary feasibility outcome of mentor recruitment rates, as outlined below.

Voluntary written or verbal informed consent and assent were obtained from caregivers and children, respectively, to participate in the study (HE 2015/240). Voluntary written informed consent was also obtained from schoolteachers, programme mentors and caregivers to participate in follow-up yarning.

In keeping with *Yuin* cultural practices and the Australian Institute of Aboriginal and Torres Strait Islander Studies (AIATSIS) ethical guidelines [35], families were also invited to attend a face-to-face information session where the details about the research and the afterschool cultural programme were presented. Participants were informed that they may consent to participate in the programme and study, or just the programme itself.

### Afterschool cultural programme and evaluation

The development of the afterschool cultural programme was informed by a formative scoping needs assessment of focus groups with children [33] and ongoing community consultation and collaboration. The academic researchers, Elders and community members, including children and caregivers, worked in partnership to co-create the programme and the evaluation components of the research. Implementing an afterschool cultural programme for primary school-aged children where children can connect with Country and learn about traditional and non-traditional cultural practices was identified by all stakeholders as a focal point for improving the health and wellbeing of the children and the communities.

The 10-week afterschool cultural programme was designed to run two afternoons per week (15:30–17:30), delivered to Aboriginal children and their siblings, by two local Aboriginal mentors (one male and one female). The mentors were employed and identified by Elders as being role-models holding appropriate cultural knowledges. In addition, visiting Aboriginal volunteers were invited to share community-specific knowledges related to programmed activities. The volunteers were compensated for their time. A programme coordinator for each community oversaw the running of the programmes. The children were provided with healthy afternoon snacks and hot meals. A research coordinator was employed to oversee the study. The programmes and evaluation were conducted between July 2016 and December 2018.

While the basic programme framework remained the same (i.e. the length and frequency of programme delivery), each community created their own agenda of activities. The agenda was designed to reinforce and build on the children’s connection to Country while promoting healthy lifestyles. Activities included locating, preparing and eating local native food (bushtucker), bushwalks, boomerang throwing, traditional games and sports, identifying bush medicine, and learning and celebrating culture through language, song and dance. In addition, the children were invited to use photography to capture what culture and Country meant to them.

Prior to commencement of the afterschool programme, health outcomes to be assessed were identified by the communities in partnership with the research team. The methods and tools to assess these health outcomes were identified through the literature. These were measured at baseline and after implementation using *validated tools* [36], validated using Western approaches. Details of the health outcomes and tools used are summarised in Table 1. The purpose of this feasibility study was not to report on the effectiveness of the cultural programme in relation to child health outcomes, rather to report the suitability and cultural appropriateness of the tools and methods used to assess the health outcomes.

**Table 1:** A summary of the participant-centred health outcomes, measures/approaches used, completion rates and the assessment time-point

Outcome	Measure/approaches	Baseline % complete data (*n*)	Follow-up % complete data (*n*)
**Demographics**			
Caregiver and family demographics	Caregiver-reported questionnaire—caregiver age, sex, Aboriginal status, Cultural and Linguistic Diversity, postcode (Index of Relative Aboriginal Socioeconomic Outcomes [37]), marital status, education level, employment status and family structure	81% (90/111)	NA
Child demographics	Caregiver-reported questionnaire—child’s age, sex and Aboriginal status	81% (90/111)	NA
Food security	Caregiver-reported 2-item survey—running out of food and unable to purchase more due to cost, and difficulty ensuring a constant food intake	81% (90/111)	NA
**Physical health**			
Adiposity (body mass index)	Child’s height and weight, BMI percentiles by gender using the World Health Organization’s BMI-for-age growth reference charts for 5–19 years [38]	91% (101/111)	85% (94/111)
Dietary intake	Child’s 24 h repeated food recalls	82% (91/111)	72% (80/111)
Physical activity and sedentary time	Accelerometry—time spent in total physical activity (minutes in light, moderate and vigorous physical activity, minutes in moderate to vigorous physical activity, minutes in sedentary behaviour) during the afterschool period	85% (94/111)	66% (73/111)
Screentime	Caregiver-reported three-item questionnaire—rules about screen entertainment, number of hours and minutes a child spends using screen entertainment on a typical weekday and weekend	81% (90/111)	63% (70/111)
Sleep	Caregiver-reported total minutes of sleep per night on a typical night	81% (90/111)	63% (70/111)
**Education**			
Afterschool activities	Caregiver-reported—“How many days of the week does your child attend an afterschool activity (e.g. homework club, sports practice and games)?” (ranging from 0 to 5 days)	81% (90/111)	63% (70/111)
School attendance	Caregiver-reported—“How many days did your child miss school in the last school term?”	81% (90/111)	63% (70/111)
**Spiritual and Social and Emotional wellbeing data**			
Cultural connectedness	Child-reported short questionnaire—“I feel good about being Aboriginal or Torres Strait Islander when I am in class” and “I enjoy sharing things about being Aboriginal or Torres Strait Islander when I am in class” (6-point Likert scale from “No: Never” to “Yes: Always”)	84% (93/111)	79% (88/111)
	Yarning circles	70% (78/111)	67% (74/111)
	Photography and yarning		Photo—70% (78/111) Yarn—55% (61/111)
Social and Emotional Wellbeing in the school and home context	Caregiver- and teacher-reported Strengths and Difficulties questionnaire	Caregiver—81% (90/111) Teacher—57% (63/111)	Caregiver—63% (70/111) Teacher—57% (63/111)

*NA* not applicable, *BMI* body mass index

### Outcome measures

Feasibility, for the purpose of this paper, refers to both feasibility of the study methods and the programme implementation, which were followed up with evaluative yarning as outlined below. The primary feasibility outcomes of mentor and child recruitment were needed to achieve the required mentor to child ratios, as outlined below. Measures of feasibility success were determined through consultation with community and/or published literature.

#### Feasibility outcomes of the study methods

(i) Recruitment rates of the mentors: the target was to recruit two mentors per community (one male and one female).
(ii) Recruitment rates of the children: the number of eligible children who enrolled in the study and/or the programme, with a maximum of 15 children to every mentor recruited [34].
(iii) Compliance in data collection: the percentage of completed outcome measures at baseline and follow-up. “Complete” was defined as 100% or partial completion of the outcome measure, while non-responses were classified as “incomplete”. Reasons for missing health and behavioural data at baseline and follow-up were also documented. Data collection was considered feasible if there was less than 20% incomplete [39].

#### Feasibility outcomes of the programme

(i) Programme attendance rates of the children: The programme mentors were trained to record the attendance at the start of each programme session to determine percentage attendance rate. A 60% attendance rate was identified as appropriate by the communities.
(ii) Retention rates of the children: the percentage of enrolled children remaining in the programme and the study at the completion, including reasons for withdrawal. An 80% retention rate was identified as a feasible outcome by the communities.
(iii) Programme delivered by mentors and guests as planned: The mentors developed a programme agenda and were trained to keep a record of what was delivered during each session including reasons for why activities were not provided or were modified. A researcher conducted direct observation of two randomly selected sessions to compare activities with the original agenda. The percentage of activities observed compared to the original agenda were reported. Programme delivery feasibility was discussed and determined with the mentors.
(iv) Child enjoyment of the programme: level of enjoyment in the programme was rated by children on a 5-point Likert scale using an age-appropriate emotional face scale ranging from 1 = did not like it at all (sad face) to 5 = I loved it (smiling face). The programme was considered feasible if at least 80% of children reported an enjoyment score of 4 or greater based on discussions with the community. Open-ended questions (“What did you like best about the programme?” and “What didn’t you like about the programme?”) were also used to identify what the children liked and disliked about the programme.

#### Demographic characteristics

Demographic characteristics of participating children and their parents/caregivers were collected using a parent/caregiver-reported survey. Parent/caregiver variables collected included parent/caregiver age, sex, Aboriginal status, marital status, educational level and current employment status. Child variables collected included age, sex and Aboriginal status. In addition, family-level variables, including family structure (primary caregiver, number of adults and children living in the house), primary language spoken at home and postcode were also assessed.

#### Follow-up yarning circles

After the programmes were completed, yarning circles were conducted separately with the children and programme mentors, caregivers and schoolteachers to obtain feedback about the feasibility of the programme and the study methods, including approaches to recruitment and retention of children; delivery and content of the programmes; appropriateness of the study methods and participant outcomes; and feedback on how the programme and study protocols could be improved. The yarning occurred outside for the children and inside for the adults. Through mentorship from the *Yuin* researcher and the local communities, the team utilised the appropriate localised cultural protocols and levels for the adults and children. Yarning circles were led by members of the research team, with some team members specifically taking note of observations from Country and participants. Journals and notes were discussed in relationship with unpacking the data from the circles and exploring key themes. The discussions were audio recorded and transcribed verbatim.

### Data analysis

All data was 100% source data verified prior to analysis [40]. In brief, a random 10% sample of data points was taken and compared with the original source (paper copy of tools). Errors in the data were corrected but the rate of error exceeded 5% hence a full review of all data and sources was completed to confirm the data quality. Quantitative data were summarised with descriptive statistics using SPSS software (version 24, SPSS, Inc., Chicago, IL). To analyse the qualitative data, an inductive thematic analysis was conducted by a non-Aboriginal and Aboriginal researcher, following the process recommended by Braun et al. [41], and confirmed by members of the Aboriginal communities. Qualitative analyses were managed in Microsoft Excel version 16 (Microsoft Corportation, USA). Both the community members and Aboriginal researcher offered critical feedback to encourage reflexivity and explore multiple/alternative interpretations that are grounded within an Indigenous standpoint [42, 43]. All data analyses were completed in consultation with the community to ensure that the Aboriginal people’s views were clearly reflected [44]. A part of this process of relationality [21] is to identify points of similarities, difference and contention and work together to find connecting and resolvable pathways.

## Results

### Research participant characteristics

A total of 111 children enrolled in the programme and consented to have their health and behavioural outcomes assessed as part of the project. A majority were boys (54%) and the average age was 8.88 years (SD = 2.15) (Table 2). Ninety-six percent of children identified as being Aboriginal. The children who did not identify as being Aboriginal were siblings of the Aboriginal children. For caregivers of the children (*n* = 90), a majority were female (77%) aged 37.09 years (SD = 9.03), ranging from 25 to 66 years. Sixty-seven percent identified as Aboriginal.

**Table 2:** Basic characteristics of participants of the project

**Child characteristics (*n* = 111)**	
Boys, *n* (%)	60 (54.05)
Girls, *n* (%)	51 (45.95)
Mean age, years (SD)	8.88 (2.15)
Indigenous status, *n* (%)	
*Aboriginal*	106 (95.50)
*Torres Strait Islander*	1 (1.00)
*None*	4 (3.60)
**Caregiver characteristics (***N*** = 90)**	
Males, *n* (%)	21 (23.33)
Females, *n* (%)	69 (76.67)
Mean age, years (SD)	37.09 (9.03)
Indigenous status, *n* (%)	
*Aboriginal*	60 (66.67)
*Torres Strait Islander*	0 (0.00)
*None*	30 (33.33)
Education, *n* (%)	
*Primary school or equivalent*	3 (3.33)
*Year 10 or equivalent*	32 (35.56)
*Year 12 or equivalent*	17 (18.89)
*Trade/apprenticeship/certificate*	22 (24.44)
*Diploma*	10 (11.11)
*University degree*	6 (6.67)
Employment, *n* (%)	
*Not employed*	45 (50.00)
*Self-employed*	3 (3.33)
*Part-time employed*	19 (21.11)
*Full-time employed*	14 (15.56)
*Other (e.g. household tasks, volunteer)*	9 (10.00)
Average number of children per household, *n* (SD)	3 (1.22)
Average number of adults per household, *n* (SD)	2 (0.83)

### Feasibility of the study methods

#### Recruitment rates of mentors and children

The total number of children who participated in the afterschool cultural programme was 120 children. The average number of children enrolled per programme was 20 children (ranging from 17 to 27 children) (Table 3). Community Elders successfully identified one male and one female mentor to run the programmes to ensure women’s and men’s business were taught appropriately. Overall, across the communities, a total of 16 mentors (7 males and 9 females) were recruited. One programme was run by two female mentors, with the male programme coordinator available to assist as required. Two programmes were run by four mentors each (two males and two females) who job shared the roles (Table 3).

**Table 3:** Recruitment, attendance, retention rates and delivery of original agenda for each programme

Programme	Recruitment of children (*n*)	Consent for health measurements (*n*)	Attendance (%)	Retention (*n*)	Delivery of original agenda (%)
Community 1					
Programme 1	17	16	64	15	100
Programme 2	21	20	71	19	83
Community 2					
Programme 3	18	18	45	16	50
Programme 4	27	26	66	25	100
Community 3					
Programme 5	17	17	74	17	83
Programme 6	20	14	61	19	0

#### Compliance in data collection at baseline and follow-up

Overall, 74.4% of all health outcome data were collected across baseline and follow-up. Of these data, 86.5% of baseline health measures and 55.9% of follow-up measures were completed (Table 3). There was no statistical difference between baseline and follow-up compliance rates. The main reasons data were incomplete (non-response) were (i) the participant was absent on the day of data collection and could not be followed up, (ii) the participant did not want to complete the assessment and (iii) technological issues of measurement tools. Further investigation into why participants did not want to complete the assessment exposed that caregivers found some of the assessments culturally inappropriate and represented a deficit viewpoint, as well as a lack of time to complete.

### Feasibility of the programme

#### Programme attendance rates of the children

The median attendance was 73% (IQR = 40%) across all six programmes (Table 3).

#### Retention rates of the children

At follow-up, 92.5% of children (*n* = 111/120 children) who were enrolled in the programme were retained (Table 3). Reasons for withdrawing included moving away from the area (*n* = 4 children), disinterest in the programme (*n* = 4 children) and one child could not be contacted.

#### Programme delivered as planned

The main reason for changes to the programme agenda by the mentors were cultural observance. For example, when Country provided a teaching opportunity, the mentors adapted the programme accordingly. The mentors who understood their cultural pedagogy recognised teachings from Country while on Country. Overall, 69% of all programme agendas were adhered to on the days of direct observation (range 0–100% adherence) (Table 3). One of the sessions observed had to be cancelled due to poor weather and no children turning up. The programmed activities for another three sessions were changed on the day. Following these sessions, the mentors stated that they wanted to do other activities based on the messages they were receiving from Country.

#### Child enjoyment of the programme

Ninety children completed the enjoyment survey (81% of consenting children) with 89% of children (80/90 children) reporting that they either “liked it” or “loved it”. One child reported that they “did not like it at all” and another child reported that they “did not really like it”. A further eight children reported that the programme “was okay”.

The key themes that arose through the open-ended enjoyment question, “What did you like best about the programme?”, pertained to “learning about culture”, “going out on Country”, “trying new things”, “meeting new people” and “sharing culture”. In response to the open-ended question, “What didn’t you like about the programme?”, majority of the children of children reported not having any dislikes regarding the programme. For those that did respond, two key themes emerged, “disrespectful behaviours of other children” and “preferences for other activities”.

### Follow-up yarning circles

Children (*n* = 74), teachers (*n* = 5), Aboriginal Education Officer (*n* = 1), programme mentors (*n* = 5) and caregivers (*n* = 4) participated in 12 yarning circles across the three communities. Three key themes emerged—feasibility of study methods, feasibility of programme design and implementation and acceptability of the programme.

#### Feasibility of study methods

Strategies for recruitment were discussed by the mentors and teachers. One group made a suggestion of using more visual strategies such as a video to assist with recruitment, “you can put together a small video clip. Like you’re talking Aboriginal learning” (Mentor). Word of mouth was identified as the most effective strategy, with more children wanting to attend by the end of the programme and an increase in numbers for the second round of programmes in each community.

“So I think at the start there was a lot of kids going ‘Oh well we don’t know what we’re gettin’ into’, you know, ‘we’re not gonna do it’. And after a couple of years the kids that did do it, talking to their friends, talking to their families and, ... we had kids that didn’t do the programme they were approaching us going ‘Oh, we wanna do it’.” (Mentor)

The recruitment methods used attracted the children who already had some level of cultural knowledge. A number of the teachers mentioned wanting to recruit the children who were not as connected with their culture and harder to reach.

“If you look at our population ...there’s about 65 Aboriginal and Torres Strait Islander students in the school, and I think there’s ... about 40 percent of those kids that we didn’t really see any interest, and ... there’s some I would’ve loved to get in there because I think they were probably more needier than the kids that we got in.” (Teacher)

One of the mentors acknowledged that recruiting children requires a relationship with the children.

“*…*knowing who these kids are, some of ‘em have got family problems, some of ‘em have got broke down family problems, some of ‘em are from single parent backgrounds, some of ‘em have got ADHD, some of ‘em have got just common health or behavioural issues. So you need to understand these kids before you can go throwing them into something. So by building the relationship with them, by communicating with them, by understanding who they are or where they’re from and what it’s all about....” (Mentor)

Caregivers, teachers and mentors reflected on the participant outcomes assessed. It was identified that some important outcomes should be included in the future to capture the “whole story”, including child confidence and maturity levels, and school-related behaviours. For example, the teachers identified that school attendance may not have changed, however, engagement in school activities and the number of negative incidents reported, such as detentions, may be a better indicator of the impact on school-related behaviours.

The adult yarning circles discussed the appropriateness of the methods to capture participant outcomes and capturing the journey of behaviour change. Strategies included better use of photography and storytelling through journaling and documenting real-time experiences to help uncover the “why” behind participant’s responses. The cultural connectedness survey was identified as not capturing cultural and localised contexts of information with the wording to be negotiated and modified for future programmes.

#### Feasibility of programme design and implementation

From the community’s perspective, involving the children in the design from the beginning and using a bottom-up approach was critical.

“So I think it was important that we had the kids, when we had the consultation with the families, the children were there with their parents. So they had consultation and a say in it from day dot. How it was gonna run, the programmes, the naming, the types of activities they wanted to learn and do, the cultural perspective, so I think it was really important that they had ownership of that, ..., I think that consultation is valuable and I’m 100 percent sure that, it gives the kids that type of leadership as well, because I know a couple of the older boys, they were quite proud to go around and promote it.” (Aboriginal Education Officer)

Mentors appreciated the ability to design and implement the programme with flexibility, in the way they wanted, giving them ownership and control. Each participating community is unique with their own cultural protocols, and this was respected when designing and implementing a programme.

“The non-pushing of certain agendas…there was no interference. ... because they were able to let the mentors and Elders run with what they were doing so that was respectful as well.” (Mentor)

While everyone supported the programme, the time of year and the days were identified as an important factor impacting attendance and types of activities implemented. Programmes during the winter months had a lower attendance rate due to poor weather, reduced sunlight and colder temperatures. These conditions impacted a number of activities, including bushwalks and traditional games, resulting in a modification or cancellation as a result. Furthermore, the days of the programme often clashed with other activities, such as sports training. Transport was identified as a key factor in attendance and retention of children and an important consideration for future programmes.

The ages of children were an important consideration in the design and the need for flexible programming. Mentors who were new to teaching found the age differences challenging in meeting the ranges of educational/pedagogical needs of the children through the programme agenda. This is another element for future programmes to be re-negotiated. Children and mentors commented about gaps in the programme content and suggested activities they would like for future programmes that could extend the children’s knowledge and connection, such as “we can make the fishing rod and then we can fish and then we can cook it on the fire” (Child) and “on the other tribes…Because they’re always wanting to find their roots” (Mentor).

#### Acceptability of the programme

There was a strong sense of acceptance of the programme among all the participants. The caregivers, teachers and mentors knew the children enjoyed the programme because “they kept coming back. If they weren’t interested, they wouldn’t have come to it” (Mentor). The mentors’ comments were supported by the dialogue of the children, noting the pride and connection to culture experienced by the participants.

“It makes me proud what I’ve learnt about my culture and how I get to learn about where I came from... now that I know things that you just taught me.” (Child)

When participants were asked what they would like to see in future programmes, not only did they mention programme content ideas, but everyone commented they wanted the programme to continue, demonstrating a strong level of acceptance.

“That we could like make like it go on for a long time.” (Child)

“I think it’s important [to keep the programme going] because they’re learning positives that go along with it....”. (Caregiver)

## Discussion

This paper reported on the feasibility of the study methods and the implementation of an afterschool programme aimed at promoting cultural connectedness and healthy lifestyles among Australian Aboriginal children and their siblings. The results showed that the communities embraced a programme that was adapted to the local context, with appropriate mentor to child ratios across all programmes being achieved. Further, a high retention rate and level of enjoyment were reached. Following discussions with the Elders and project stakeholders, the attendance rates were appropriate to the communities. Potential targets for participation, recruitment, retention and compliance within the programmes and specific areas for improvement were identified. A relational methodology, inclusive of co-created mixed methods, was vital to programme acceptance and gaining a deeper understanding of the key feasibility findings.

As the study focused on Aboriginal communities, it was imperative to examine the concept of feasibility through Country and the lens of ARRM. Country is a living entity that is a teacher and holds knowledge [1, 26, 45]. If we observe Country, we can be guided in our understanding of the concepts through the data [1, 45]. When Country from an Aboriginal standpoint is placed back into being the central tenet in knowing, doing and being then the findings are guided by Country through people (us the researchers) knowing and engaging in this knowledge system. Employing this approach created cultural and knowledge tensions with academic structures and research approaches and challenged our understanding, application and measurement of feasibility. The research exposed a disjunction between Western feasibility constructs and understanding feasibility using ARRM.

The community comprehends feasibility differently from academia. For example, the community recognised the feasibility of Country (Country as teacher) changing the programme teaching content, whereas the Western understanding would state that the programme did not follow the set curriculum and therefore was not delivered as planned and categorised as poor feasibility. As such, we are trying to find points of connection between a Western understanding of feasibility and an Indigenous understanding of feasibility, thus finding a middle ground where both understandings are respected. Bamblett et al. [36] has found that this is common when working in two knowledge systems but highlights that even though these two knowledges of feasibility appear contradictory, they can actually be complementary and provide a holistic view of programme feasibility. To be inclusive of this holistic view, First Nation approaches can be utilised. As a Canadian First Nation academic, Wilson [21] states “there is no need to be critical of or judge others’ ideas or theories if all are thought of as equally valid. Rather there is a need for each person to develop his or her own relationship with ideas and to therefore form their own conclusion” (p. 94). It is therefore important to move beyond Western-only frameworks and acknowledge the value of both knowledge systems to obtain a more in-depth, holistic understanding of programme and study method feasibility within Aboriginal contexts [36, 46, 47].

In the current study, feasibility within the Aboriginal community context was repositioned in relationship with Western understandings: not separated [30, 36]. If we interpret the findings solely through a Western lens predominantly led by humans, the quantitative feasibility measures in isolation may be considered “weak”. However, the application of the qualitative Aboriginal elements within the evaluation and inclusion of Country in interpreting findings provided a holistic evaluation of the programme, demonstrating acceptance and feasibility within the community. We are not saying that quantitative measures cannot be used, we are saying that it is important to use both relational approaches and quantitative outcomes to gain a holistic understanding of the context. For instance, 69% of the programmed activities were delivered as planned but through an Indigenous lens where Country leads the programme, 100% was delivered as planned. The Aboriginal mentors listened to the messages from Country (Country as curricula) and adapted the programme activities according to what Country was teaching. By way of example, one planned activity was to take children on a bushwalk to the river. However, on the way to the river, the group came across a number of Willy Wagtail birds and the teaching changed to a teaching around the birds’ story. The group never made it to the river because Country presented a teaching opportunity. *Yuin* Country as teacher is a space and place that produces messages and teaching from non-human entities and understandings are developed through feelings in regard to the cultural learning experience [1]. This demonstrates the importance of two knowledge systems working together to understand feasibility [36].

In adopting ARRM, informed by Country, our feasibility assessment has highlighted three interconnected important considerations for future iterations of the project, namely (i) strong partnerships for co-creation; (ii) embracing flexible methodology; and (iii) suitability of methods.

### Strong partnerships for co-creation

The partnerships and co-creation process were critical for guiding the development, implementation, evaluation and contribution to the feasibility and acceptance of the afterschool cultural programme by the community [46, 48]. Historically and today, many Aboriginal health programmes have been developed without active involvement from the participants that the programme was designed for [49]. Demonstrated in the feasibility of our programme design and implementation results, the community members welcomed and recognised the value of consultation with all participants, especially the children, having an opportunity to voice their learning experiences and incorporate cultural protocols. The value of this may have contributed to the targeted and successful recruitment, high retention rates of children and enjoyment of the programme. By way of example surrounding cultural protocols, one element that was imperative across all three communities was the need for female and male mentors to meet cultural protocols on girl’s and boy’s business to educative practice. There was a need to encompass an Aboriginal pedagogical, healing and educational approach by the mentors when working with Country, and these skills were strengths in guiding the children. Furthermore, in defining feasibility measures and outcomes using ARRM, it is important to recognise that not adopting a partnership approach may lead to unsustainable and ineffective outcomes that perpetuate the historically known damage from Western research practices [23, 46].

The communities’ voices and the AARM literature helped the research team to become more aware of the tensions and contestations arising from the partnership. Through communication and negotiations, specific roles and responsibilities were identified to reduce potential interference of cultural practice/methods. More specifically, the project team with the community worked through communication on tensions and protocols from both knowledge systems, towards finding a point of understanding in identifying responsibilities, while not interfering with each other’s duty. For example, the community were leaders in implementing and teaching the programme, while the research team worked through the tensions around the methods and implementing the methods to obtain data from the programme. Through ARRM, visits on Country together, and observing each other during the programme, time was taken to identify each other’s strengths and how they were positioned within the relationship, programme and the overall project. This enabled the project team to develop a shared equitable leadership approach during different phases of the research to enhance the feasibility of the project [36].

### Embracing flexible and culturally embedded methodologies

Flexibility over the course of the project became a key priority identified through ongoing community consultation and our own reflections of the programme and data collection. As stated above, embracing a flexible two-way knowledge system respectfully to reduce colonial residues was essential. While the underlying framework remained the same across each community, a flexible methodology acknowledging the different localised knowledge systems contributed to the feasibility and acceptance of the afterschool cultural programme within each community [36, 46, 48]**.**

Flexibility of the programme and project was instilled through Country being a central focal point [30] and our growing understanding and implementation of ARRM [26]. This evolving understanding of knowledge invited the team to reflect on Western research norms and how these fitted within culturally embedded methodologies throughout the project (i.e. through the design, implementation and evaluation phases). The design phase needed to be flexible and adaptive to each community and Country, which was a core requirement to sustaining, enabling and empowering the community, mentors and children [46]. The project demonstrated moderate acceptance and compliance rates in relation to the health measures; however, this was subject to the presence of flexibility and adaptability within its design. We acknowledge that not all health measures could be flexible in regard to how the measures were delivered and data gathered. However, flexibility could be present in the interpretation of the data which needed to be inclusive of cultural understandings from both knowledge systems without valuing one over the other (the tensions between Western and Aboriginal knowledge systems). A “one size fits all” approach and design would have impeded and disempowered the children’s connection to Country and have continued colonial practice [30, 36]. ARRM allowed for an understanding of *why* a decline in the compliance of health measures occurred enabling the researchers to reflect on the appropriateness of the tools that were selected.

Finally, the research team had to adopt the ARRM approach in an attempt to decolonise “accepted” research approaches in academia, and be flexible around the methods to ensure the communities’ and academic needs were met [48]. Flexibility was also important to ensure workable, respectful relationships, with a shared goal to connect children to their culture, and enhance health and wellbeing.

### Suitability of methods

During the design phase of the study, tools were identified through the literature, some of which were adapted to Indigenous populations. Despite these tools and methods having certain strengths (such as statistical analysis, psychometrics and various discipline of interpretation and conceptualisation), it was not until the implementation and interpretation of the data that the team noticed a disconnect between the tools and Aboriginal ways of doing, being and knowing. It became clear that the tools do not take into account culture, beliefs, connection to community and place, spirituality and individual experiences, which are key constructs within the ARRM methodology. Even though some of the tools had been adapted to Indigenous population, they are still susceptible to and maintain Western cultural bias reinforcing the deficit and colonial perspectives and practises [36, 48, 50]. This was demonstrated in the low completion rates of certain tools that had been validated in Indigenous populations, e.g. the Strengths and Difficulties questionnaire [51]. In adopting the relational approach and strengthening the relationships with the communities over the course of the project, the Aboriginal community identified that these tools were not appropriate as it reinforced the deficit and stereotypical colonial view of health and of Aboriginal peoples.

As an alternative, there was a shift towards adopting culturally appropriate, strengths-based approaches. In examining culturally appropriate, strengths-based approaches, Fogarty et al. [50] stated that importance of adopting these approaches because they “can be a highly effective method for shifting or changing narratives of Indigenous health, and to illuminate and provide alternative ways to deal with health issues effecting Indigenous peoples” (p.29). As such, in establishing the suitability of methods, the research team listened to the communities and engaged in continual negotiation and evaluation. This resulted in the addition of more culturally appropriate methods and tools that were based on cultural understanding, cultural respect and community engagement (e.g. storytelling, photography, and yarning) [1, 48, 52, 53].

Upon reflection, the research team also acknowledge that not all tools used in this current feasibility trial have been decolonised and this is an area for continual (re)development, (re)negotiation and (re)consideration for future programme evaluations [54, 55]. This highlights the importance for similar studies using a relational flexible methodology. This works to actively reinforce the importance of the community and Country informing the research at all phases [55].

### Strengths and limitations

One of the strengths of this study was the relational methodology used from the conception of the project through to the community feedback stage. This ensured that the project was culturally grounded with the end goal of meeting the needs of all project stakeholders. Another strength of the study was the time taken to build and grow the relationships ensuring that each stage of project evolution was agreed upon as a collective.

This research was not without limitations. As a feasibility study, the sample was not intended to be generalisable or hold sufficient statistical power of the effectiveness of the programme on participant outcomes. Some methods selected were not deemed to be feasible yet were maintained in different forms to maintain data integrity. While this allowed for reporting of health behaviours, the tools used were deemed unsuitable. New tools and methods for future research will need to be negotiated, implemented and reviewed with community to allow for respectful two-way processes for the project to continue [55].

## Conclusion

While it has been known for centuries that having a strong cultural identity and connection to Country has enabled Aboriginal people to have a healthy lifestyle [3], this study was a vital first step in determining the potential sustainability of an Aboriginal afterschool cultural programme in promoting health and wellbeing through cultural connectedness among Aboriginal children. Through the use of mixed methods, the study found that a programme needs to be adaptable to the local context encompassing flexibility in its design through listening to Country and the community voices. The findings of this feasibility study and the identified areas for improvement will be instrumental to future iterations of the afterschool cultural programme [5]. Future research should examine the transferability of the programme to other settings, such as high schools and other community settings yet be grounded in relational methodologies. Future programmes with Australian Aboriginal communities need to be built on strong partnerships and embrace a flexible culturally embedded methodology, such as ARRM, in order to be adaptive and responsive to research approaches, communities and to Country.

Overall, the programme was considered feasible by the communities due to the high participation, enjoyment, recruitment and retention rates. Some of the study methods tested within this feasibility study were not deemed feasible and need to be re-negotiated with the community in future iterations of the programme. These feasibility targets were established through continual negotiation with the communities and consideration of Country across all stages of the research.

## Data Availability

The datasets generated and analysed during the current study are not publicly available due to the request from Cullunghutti Aboriginal Child and Family Centre to protect data from inappropriate use but are available from the corresponding author on reasonable request with permission from Cullunghutti Aboriginal Child and Family Centre. In appreciation and recognition to Cullunghutti Aboriginal Child and Family Centre and the local Shoalhaven Aboriginal communities, Cullunghutti Aboriginal Child and Family Centre asks that this material is not reproduced without permission and is treated with dignity and respect.

## Abbreviations

AIATSIS: Australian Institute of Aboriginal and Torres Strait Islander Studies
ARRM: Aboriginal Relational Research Methodology
BMI: Body mass index
IQR: Interquartile range
IRM: Indigenous research methodology
NSW: New South Wales
SD: Standard deviation
n: Number

## References

[1] McKnight A (2017). Singing up country in Academia: Teacher education academics and preservice teachers’ experience with Yuin Country.

[2] Kwaymullina A (2005). Seeing the light: Aboriginal law, learning and sustainable living in country. Indig Law Bull.

[3] Waterworth P, Pescud M, Braham R, Dimmock J, Rosenberg M (2015). Factors Influencing the Health Behaviour of Indigenous Australians: Perspectives from Support People. PLoS One.

[4] Dockery AM (2020). Inter-generational transmission of Indigenous culture and children’s wellbeing: Evidence from Australia. Int J Intercult Relat.

[5] Ganesharajah C. Indigenous health and wellbeing: the importance of country, Native Title Research Unit, Australian Institute of Aboriginal and Torres Strait Islander Studies, Canberra, 2009.

[6] Houston S (1989). National aboriginal health strategy working party. Aborig Isl Health Work J.

[7] Gee G, Dudgeon P, Schultz C, Hart A, Kelly K. Aboriginal and Torres Strait Islander social and emotional wellbeing. In: Working together: Aboriginal and Torres Strait Islander mental health and wellbeing principles and practice. Vol. 2. 2014. pp. 55-68.

[8] Garvey G, Anderson K, Gall A, Butler TL, Whop LJ, Arley B (2021). The Fabric of Aboriginal and Torres Strait Islander Wellbeing: a conceptual model. Int J Environ Res Public Health.

[9] Garvey G, Anderson K, Gall A, Butler TL, Cunningham J, Whop LJ (2021). What Matters 2 Adults (WM2Adults): Understanding the Foundations of Aboriginal and Torres Strait Islander Wellbeing. Int J Environ Res Public Health.

[10] Garnett S, Sithole B (2008). Sustainable northern landscapes and the nexus with Indigenous health: Healthy Country, healthy people: Citeseer.

[11] Willis E, Pearce M, Jenkin T (2004). The demise of the Murray River: insights into lifestyle, health and well-being for rural Aboriginal people in the riverland. Health Sociol Rev.

[12] Burgess CP, Johnston FH, Berry HL, McDonnell J, Yibarbuk D, Gunabarra C (2009). Healthy Country, healthy people: the relationship between Indigenous health status and “caring for Country”. Med J Aust.

[13] Priest N, Mackean T, Davis E, Waters E, Briggs L (2012). Strengths and challenges for Koori kids: Harder for Koori kids, Koori kids doing well–Exploring Aboriginal perspectives on social determinants of Aboriginal child health and wellbeing. Health Sociol Rev.

[14] Salmon M, Doery K, Dance P, Chapman J, Gilbert R, Williams R (2018). Defining the indefinable: Descriptors of Aboriginal and Torres Strait Islander Peoples’ cultures and their links to health and wellbeing.

[15] Schultz R, Cairney S (2017). Caring for country and the health of Aboriginal and Torres Strait Islander Australians. Med J Aust.

[16] Bullen J, Hill-Wall T, Anderson K, Brown A, Bracknell C, Newnham EA (2023). From deficit to strength-based Aboriginal health research—moving toward flourishing. Int J Environ Res Public Health.

[17] Lines L-A, Jardine CG (2019). Connection to the land as a youth-identified social determinant of Indigenous Peoples’ health. BMC Public Health.

[18] Sorensen G, Emmons K, Hunt MK, Johnston D (1998). Implications of the results of community intervention trials. Annu Rev Public Health.

[19] Potvin L, Cargo M, McComber AM, Delormier T, Macaulay AC (2003). Implementing participatory intervention and research in communities: lessons from the Kahnawake Schools Diabetes Prevention Project in Canada. Soc Sci Med.

[20] Johnstone M-J (1991). Improving the ethics and cultural suitability of Aboriginal health research: some further suggestions. Aborig Isl Health Work J.

[21] Wilson S. Research is ceremony: Indigenous research methods. Nova Scotia: Fernwood publishing; 2008.

[22] Harfield S, Pearson O, Morey K, Kite E, Canuto K, Glover K (2020). Assessing the quality of health research from an Indigenous perspective: the Aboriginal and Torres Strait Islander quality appraisal tool. BMC Med Res Methodol.

[23] Pidgeon M, Riley T (2021). Understanding the Application and Use of Indigenous Research Methodologies in the Social Sciences by Indigenous and Non-Indigenous Scholars. Int J Educ Policy Leadersh.

[24] Dudgeon P, Bray A, Darlaston-Jones D, Walker R. Aboriginal participatory action research: an Indigenous research methodology strengthening decolonisation and social and emotional wellbeing: The Lowitja Institute; 2020.

[25] Pyett P, Waples-Crowe P, Van der Sterren A (2008). Challenging our own practices in Indigenous health promotion and research. Health Promot J Austr.

[26] McKnight ADB, Probst Y, O'Flynn G, Tillott S, Stanley RM. Relationships are essential but not always easy: The role of methodology in embedding Aboriginal community and Country in academic research. Health Promot J Austr. 2023. 10.1002/hpja.781. 10.1002/hpja.781 37491724

[27] Country B, Wright S, Suchet-Pearson S, Lloyd K, Burarrwanga L, Ganambarr R (2016). Co-becoming Bawaka: Towards a relational understanding of place/space. Prog Hum Geogr.

[28] Martin K. Please knock before you enter: Aboriginal regulation of outsiders and the implications for researchers: Post Pressed; 2008.

[29] Bunda T, Zipin L, Brennan M (2012). Negotiating university ‘equity’from Indigenous standpoints: a shaky bridge. Int J Incl Educ.

[30] McKnight A (2015). Mingadhuga Mingayung: respecting Country through mother mountain’s stories to share her cultural voice in western academic structures. Educ Philos Theory.

[31] Nakata M (2007). The cultural interface. Austr J Indig Educ.

[32] Bhabha HK. The location of culture: routledge; 2012.

[33] Crowe R, Stanley R, Probst Y, McMahon A (2017). Culture and healthy lifestyles: a qualitative exploration of the role of food and physical activity in three urban Australian Indigenous communities. Aust N Z J Public Health.

[34] Australian Children’s Education and Care Quality Authority. Guide to the National Quality Framework. 2020. Available from: https://www.acecqa.gov.au/sites/default/files/2020-01/Guide-to-the-NQF_2.pdf.

[35] Australian Institute of Aboriginal Torres Strait Islander Studies (2003). Guidelines for ethical research in Indigenous studies. Austr Indig Law Rep.

[36] Bamblett M, Frederico M, Harrison J, Jackson A, Lewis P (2012). Not one size fits all: Understanding the social and emotional wellbeing of Aboriginal children.

[37] Biddle N (2009). Ranking regions-revisiting an index of relative Indigenous socio-economic outcomes. Aust J Reg Studi The.

[38] Onis Md, Onyango AW, Borghi E, Siyam A, Nishida C, Siekmann J. Development of a WHO growth reference for school-aged children and adolescents. Bulletin of the World health Organization. 2007;85(9):660–7. 10.2471/BLT.07.043497 PMC2636412 18026621

[39] Mason MJ (1999). A review of procedural and statistical methods for handling attrition and missing data in clinical research. Meas Eval Couns Dev.

[40] Houston L, Probst Y, Humphries A (2015). Measuring data quality through a source data verification audit in a clinical research setting. Stud Health Technol Inform.

[41] Braun V, Clarke V, Terry G, Rohleder P, Lyons A (2014). Qualitative research in clinical and health psychology.

[42] Fletcher A (2019). An invited outsider or an enriched insider? Challenging contextual knowledge as a critical friend researcher.

[43] Smith B, McGannon KR (2018). Developing rigor in qualitative research: Problems and opportunities within sport and exercise psychology. Int Rev Sport Exerc Psychol.

[44] Castleden H, Morgan VS, Lamb C (2012). “I spent the first year drinking tea”: Exploring Canadian university researchers’ perspectives on community-based participatory research involving Indigenous peoples. Can Geogr/Le Géographe canadien.

[45] Spillman D, Wilson B, Nixon M, McKinnon K (2023). Reinvigorating Country as teacher in Australian schooling: beginning with school teacher’s direct experiences, ‘relating with Country’. Curriculum Perspectives.

[46] Dickerson D, Baldwin JA, Belcourt A, Belone L, Gittelsohn J, Keawe’AimokuKaholokula J (2020). Encompassing cultural contexts within scientific research methodologies in the development of health promotion interventions. Prev Sci.

[47] Bodkin-Andrews G, Whittaker A, Harrison N, Craven R, Parker P, Trudgett M (2017). Exposing the patterns of statistical blindness: Centring Indigenous standpoints on student identity, motivation, and future aspirations. Aust J Educ.

[48] Finlay SM, Canuto K, Canuto K, Neal N, Lovett RW (2021). Aboriginal and Torres Strait Islander connection to culture: building stronger individual and collective wellbeing. Med J Aust.

[49] McPhail-Bell K, Bond C, Brough M, Fredericks B (2015). ‘We don’t tell people what to do’: ethical practice and Indigenous health promotion. Health Promot J Austr.

[50] Fogarty W, Lovell M, Langenberg J, Heron M-J. Deficit discourse and strengths-based approaches. Changing the Narrative of Aboriginal and Torres Strait Islander Health and Wellbeing Melbourne: The Lowitja Institute. 2018.

[51] Williamson A, McElduff P, Dadds M, D'Este C, Redman S, Raphael B (2014). The Construct Validity of the Strengths and Difficulties Questionnaire for Aboriginal Children Living in Urban New South Wales, Australia. Aust Psychol.

[52] Bessarab D, Ng'Andu B (2010). Yarning about yarning as a legitimate method in Indigenous research. Int J Crit Indig Stud.

[53] Yunkaporta T, McGinty S (2009). Reclaiming Aboriginal knowledge at the cultural interface. Austr Educ Res.

[54] Productivity Commission (2020). A guide to evaluation under the Indigenous Evaluation Strategy.

[55] Kelaher M, Luke J, Ferdinand A, Chamravi D, Ewen S, Paradies Y (2018). An evaluation framework to improve Aboriginal and Torres Strait Islander health.

